# Integrating methadone into primary care settings in Ukraine: effects on provider stigma and knowledge

**DOI:** 10.1002/jia2.26202

**Published:** 2024-02-20

**Authors:** Daniel J. Bromberg, Eteri Machavariani, Lynn M. Madden, Konstantin Dumchev, Katherine LaMonaca, Valerie A. Earnshaw, Iryna Pykalo, Myroslava Filippovych, Marwan S. Haddad, Sergii Dvoriak, Frederick L. Altice

**Affiliations:** ^1^ Yale School of Public Health, Yale University New Haven Connecticut USA; ^2^ Center for Interdisciplinary Research on AIDS, Yale University New Haven Connecticut USA; ^3^ Yale School of Medicine, Yale University New Haven Connecticut USA; ^4^ APT Foundation New Haven Connecticut USA; ^5^ Ukrainian Institute on Public Health Policy Kyiv Ukraine; ^6^ University of Delaware Newark Delaware USA; ^7^ Center for Key Populations, Community Health Center, Inc. Middletown Connecticut USA

**Keywords:** stigma, people who inject drugs, HIV, opioid use disorder, methadone, integrated care

## Abstract

**Introduction:**

Stigma has undermined the scale‐up of evidence‐based HIV prevention and treatment. Negative beliefs influence clinicians’ discriminatory behaviour and ultimately have wide‐ranging effects across the HIV prevention and treatment continuum. Stigma among clinicians can be mitigated in several ways, including through interpersonal contact. In this study, we test whether interactions with people who inject drugs (PWID) influence attitudes of both direct and indirect providers of opioid agonist therapies (OATs) within the same primary care clinics (PCCs) where OAT is newly introduced.

**Methods:**

In a cluster randomized controlled trial integrating OAT and HIV care into PCCs in Ukraine, clinicians at 24 integrated care sites (two sites in 12 regions) from January 2018 to August 2022 completed a structured survey at baseline, 12 and 24 months. The survey included feeling thermometers and standardized scales related to clinician attitudes towards patients and evidence‐based care. Nested linear mixed‐effects models were used to examine changes in mean scores over three timepoints for both direct and indirect clinicians.

**Results:**

There were fewer significant changes in any of the scales for direct providers (*n* = 87) than for indirect providers (*n* = 155). Direct providers became less tough‐minded about substance use disorders (*p* = 0.002), had less negative opinions about PWID (*p* = 0.006) and improved their beliefs regarding OAT maintenance (*p*<0.001) and medical information (*p* = 0.004). Indirect providers reported improvements in most stigma constructs, including a significant decrease in prejudice (*p*<0.001), discrimination (*p* = 0.001), shame (*p* = 0.007) and fear (*p* = 0.001) towards PWID.

**Conclusions:**

Integrating OAT services within primary settings was associated with significantly reduced stigma constructs and improved attitudes towards PWID, possibly through increased intergroup contact between PWID and general clinical staff. Unlike most stigma reduction interventions, re‐engineering clinical processes so that PWID receive their care in PCCs emerges as a multilevel stigma reduction intervention through the integration of specialized services in PCCs. Integration influences different types of stigma, and has positive effects not only on health outcomes, but also improves clinician attitudes and efficiently reduces clinician stigma.

## 1 INTRODUCTION

The topic of stigma in healthcare settings has attracted enormous attention in the public health literature, driven by the understanding that it undermines the quality of care and exacerbates individual and public health problems. Stigma is a social determinant of health that is governed by societal norms and that dictates attitudes towards certain, often marginalized groups of people [1]. Stigma exacerbates health problems at the individual level, including increasing psychological distress [2] and decreasing health‐related quality of life [3], increasing symptoms and decreasing overall health, and delaying treatment‐seeking behaviour [4]. Stigma also exacerbates substance use and psychiatric disorders in people with other diseases [4]. When healthcare workers hold stigmatizing beliefs about their patients, both the quality of and patient engagement in care is reduced in people with HIV (PWH), psychiatric disorders, and other conditions [5, 6, 7].

For people who inject drugs (PWID), stigma negatively influences the scale‐up of evidence‐based treatments [8]. PWID are heavily stigmatized, including in healthcare settings [9, 10]. Opioid agonist therapies (OATs) using maintenance with methadone or buprenorphine are evidence‐based treatments for opioid use disorder (OUD). OATs are effective as primary and secondary HIV prevention [11], improving the HIV treatment cascade [12, 13], and improving primary care outcomes [14, 15]. Despite OAT rarely being diverted and used to precipitate euphoria, some healthcare providers hold stigmatizing beliefs about OAT and people prescribed OAT [16, 17]. Negative beliefs regarding OAT can influence providers’ discriminatory behaviour and, ultimately, have wide‐ranging effects across the spectrum of patient healthcare experiences, including willingness to seek care, adherence to treatments, health outcomes and overall quality of life [18, 19].

Stigma among providers can be mitigated in several ways, including through interpersonal contact and increased knowledge [20]. In particular, interpersonal contact with stigmatized groups, such as people with mental illness, has been shown to reduce prejudice among providers [21]. A large body of literature on intergroup contact theory examines attitudes and relations between members of different groups and emphasizes the critical role of intergroup contact for strengthening communities and reducing prejudice and discrimination [22, 23, 24, 25]. Intergroup contact theory studies in healthcare, however, are usually limited to those where intergroup contact happens during a workshop, or the provider group is temporarily placed in the environment where care is provided for the stigmatized group (e.g. medical students clerkship placement at the psychiatric hospital [26]). Studies exploring contact in healthcare settings are scarce, especially those where an integrated service is introduced for a stigmatized group, relocating their care from a specialized clinic to a less stigmatizing environment (e.g. primary healthcare centres). Stigma is a complex, multilevel phenomenon, meaning that it operates on intrapersonal, interpersonal, organizational and structural levels [27]. Thus, interventions that introduce intergroup contact through re‐engineering the way services are delivered, how patients are treated or those that target structured and multilevel processes can affect stigma across multiple levels [28, 29]. Systematic reviews suggest that the benefits of multi‐level stigma interventions are likely to be most potent [28].

As part of a prospective, cluster, randomized controlled trial to compare the integration of OAT into primary care clinics (PCCs) to standard of care in specialty clinics (SOC) that specifically provide treatment for OUD, we integrated OAT into PCCs in 12 regions in Ukraine. Here, we assess prejudice towards PWID by OAT providers within PCCs at baseline (before the contact), and at 12‐ and 24‐month follow‐up time points to assess the response by providers after direct and indirect contact with PWID, with the hypothesis that the types of interactions with PWID—positive or negative—would influence changes in these attitudes by clinical providers at PCCs who provide care directly (i.e. see the OAT patients regularly) and indirect (other clinicians in the same clinic but do not provide direct clinical services) care.

## 2 METHODS

### 2.1 Study design

The present paper is a secondary analysis of the prospective, randomized controlled trial conducted in Ukraine to integrate OAT into primary healthcare (PCC) clinics and test the effectiveness of intervention against SOC, defined as OAT provided at specialty OAT treatment centres. This study involved the first integration of methadone into PCCs, incorporating lessons from an initial pilot study [30]. A cluster of two PCCs (intervention) and one SOC (control) were selected in 12 regions (Rivne, Dnipro, Zhytomyr, Sloviansk, Kyiv, Kropyvnytskyi, Kryvyi Rih, Mariupol, Mykolaiv, Kramatorsk, Cherkasy and Odesa), for a total of 24 PCCs and 12 SOC sites. PCCs were further divided into those that provided financial incentives to clinicians, pay‐for‐performance (P4P), linked to achieving pre‐defined patient outcomes versus those that provided no P4P. Eligibility criteria included age above 18 years, having a confirmed OUD diagnosis and residing within the catchment care of the clinics. After consent and before randomization, eligible participants were informed they would be randomized 2:1 to either PCCs or SOCs. Randomization was stratified 1:1 based on whether the participant was stably induced (>3 months) or new to methadone. Participants newly starting methadone received daily observed dosing, while those who had been stable on methadone for 6 months or more could receive take‐home dosing for 7–10 days.

Clinical staff providing direct OAT care at each of the 24 PCC sites were selected by their site director based on their interest and willingness in providing services to PWID (i.e., motivated to provide the care). After a 3‐day training on OAT, they received weekly ECHO‐based tele‐education on OAT, HIV, tuberculosis (TB), and quality improvement to support clinical skills in providing specialty services. Project ECHO is an innovative collaborative learning approach that facilitates knowledge exchange between experts (substance use specialists) and non‐experts (primary healthcare providers) to ensure high‐quality care delivery in non‐specialty settings (PCC) [31, 32, 33]. In other settings, it has been engineered to address stigma [34]. Clinicians at SOCs and at PCCs who were not directly involved in OAT did not receive any kind of training or intervention. For the present analysis, we only use data from PCCs as the SOC was the control group, no intervention was given to this group, and provider stigma measures were not assessed. Data for the present paper were collected between 20 January 2018 and 29 August 2022.

### 2.2 Ethics and consent

This study was reviewed and approved by the institutional review boards at Yale University (IRB #2000020067) and the Ukrainian Institute on Public Health Policy (IRB #00007612). All participants provided written, informed consent.

### 2.3 Data collection and sample

Data were collected through an online link only with PCC providers in Russian or Ukrainian, based on participant preference, using structured surveys at baseline, 12 and 24 months. Purposive recruitment included all direct OAT providers and, among non‐OAT providers, they were selected randomly from the list of all PCC clinical staff. Approximately 20 providers were surveyed at each PCC at each time point, where approximately a quarter of the sample in each clinic were direct OAT providers. Direct providers were defined as primary healthcare providers (doctors, nurses, and social workers) directly involved in OAT provision. Indirect providers were defined as clinicians (doctors, nurses, and administrators) who were employed at the same PCC where direct providers worked, but were not directly involved in OAT delivery. Indirect providers did not have any contact with patients receiving OAT in any professional capacity and their interaction was limited to casual encounters in common areas like waiting rooms and hallways which were not measured or quantified.

### 2.4 Measures

The structured survey included feelings thermometers (i.e., prejudice) towards groups of people with a range of socio‐demographic and health conditions, such as PWH, PWID, men who have sex with men (MSM), women who engage in sex work, and recently released prisoners [18, 35–39]. These were collected to examine how clinicians felt about key populations generally. Feelings thermometers were scored on a scale of 0–100, with higher scores denoting better attitudes towards the aforementioned groups. The Counsellor Assessment Screen (CAS) [40] was used to measure attitudes towards patients and methadone treatment. CAS is a validated instrument used internationally to assess the impact of provider beliefs on treatment for substance use disorders (SUDs). Four subscales included being tough‐minded about SUDs (11 items, α = 0.71), being oriented towards opioid abstinence versus maintenance treatment (22 items, α = 0.87), having negative opinions about patients (4 items, α = 0.26) and reporting incorrect medical information about SUD treatment (5 items, α = 0.80). The items included statements like “People who become addicted to heroin have only themselves to blame,” or “methadone maintenance can cause liver damage,” for example, and were scored using a 5‐point Likert scale and the total subscale score was calculated by adding the individual item scores, with higher scores denoting poor attitudes. The questions that were positively framed were reverse‐scored in the total score calculation.

Stigma was measured using a multidimensional scale adapted to assess stigma towards PWID [41]. This scale has been used to evaluate stigma towards different marginalized populations, like transgender patients, MSM and PWH [18, 35, 42]. This scale includes five subscales on discrimination (4 items, α = 0.90), prejudice (4 items, α = 0.58), internal shame (2 items, α = 0.53), fear (3 items, α = 0.81) and stereotypes (4 items, α = 0.75) towards PWID. The survey included items like “I am willing to work with injection drug users” and “injection drug users should blame themselves for their condition” to evaluate providers’ attitudes towards PWID. The items were scored on a 5‐point Likert scale and the total score for each subscale was a sum of individual item after reverse‐scoring positively framed items. Higher scores denote higher stigma. Finally, individual resistance to change was measured using a 17‐item Resistance to Change (RC) scale. The scale showed good internal consistency (α = 0.87) [43].

The scores of CAS and stigma subscales, as well as the RC scale, were standardized between 1 and 5, with higher scores corresponding to more negative attitudes. Other questions pertained to socio‐demographic characteristics and some scale items were adapted for cultural context.

Most of the scales have not been formally validated in Ukrainian and/or Russian; however, we did conduct translation/back translation of all the scales during repeated pilot testing of the survey measures and they have been used in other studies in Ukraine.

### 2.5 Data analysis

In the analytical sample, we included only those providers who had completed all three interviews to enable us to accurately follow the dynamics of within‐person attitude changes. Providers who did not complete either one or both follow‐up interviews were excluded from the analysis.

First, we performed a descriptive analysis of the sample. Then, standardized scale scores were calculated for each individual provider for all three timepoints (baseline, 12 and 24 months). For every scale, a nested linear mixed‐effects model was used to examine changes in mean scores over three timepoints for direct and indirect providers. In each model, individual providers were nested in clinics, and clinics were nested within regions, with the time variable being the main covariate. Restricting the sample to participants with complete data allowed the model to account for within‐subject correlation. Residuals of each model were visually inspected for normality and homoscedasticity. Analyses were performed using R version 4.2.1.

### 2.6 Role of the funding source

The funder of the study had no role in study design, data collection, data analysis, data interpretation or writing of the report. All authors had full access to all the data in the study and had final responsibility for the decision to submit for publication.

## 3 RESULTS

We obtained data for 242 PCC providers at baseline, 12 and 24 months that completed all three interviews. Those excluded did not differ from the original sample based on the demographic characteristics. The sample included 87 (36.0%) direct and 155 (66.0%) indirect providers. Most participants were women (85.1%), in their mid‐40s (mean = 46.1; SD 12.2) years, and had worked in their respective clinic for 15.6 years (SD 12.1), with no major difference between the two groups of providers. Approximately half of providers (51.2%) were nurses, followed by doctors (33.9%), and administrative and other staff (14.9%). The number of respondents at each of the 12 clinics ranged between 14 and 33. At baseline, over half of providers (56.5%) reported having known someone in their personal lives who was a PWID (Table 1). The number of patients receiving OAT in each clinic varied between 8 and 61 (Table S1).

**Table 1:** Primary healthcare provider characteristics

Characteristic	All providers *N* = 242	OAT providers *N* = 87	Non‐OAT providers *N* = 155
	*N* (%)	*N* (%)	*N* (%)
***Position***			
Doctor	82 (33.9)	37 (42.5)	45 (29.0)
Nurse	124 (51.2)	29 (33.3)	95 (61.3)
Other	36 (14.9)	21 (24.1)	15 (9.7)
Female	206 (85.1)	65 (74.7)	141 (91.0)
Age (mean ± SD)	46.1 (12.2)	47.8 (11.8)	45.2 (12.4)
Years at clinic (mean ± SD)	15.6 (12.1)	13.5 (11.2)	16.7 (12.4)
Personal experience^a^	137 (56.5)	61 (70.1)	76 (49.0)
***Region***			
Rivne	22 (9.1)	7 (8.0)	15 (9.7)
Dnipro	16 (6.6)	8 (9.2)	8 (5.2)
Zhytomyr	18 (7.4)	4 (4.6)	14 (9.0)
Slovyansk	23 (9.5)	8 (9.2)	5 (9.7)
Kyiv	12 (5.0)	7 (8.0)	5 (3.2)
Kropyvnytskyi	17 (7.0)	9 (10.3)	8 (5.2)
Kryvyi Rig	14 (5.8)	7 (8.0)	7 (4.5)
Mariupol	32 (13.2)	9 (10.3)	23 (14.8)
Mykolaiv	33 (13.6)	8 (9.2)	25 (16.1)
Kramatorsk	21 (8.7)	9 (10.3)	12 (7.7)
Cherkassy	14 (5.8)	8 (9.2)	6 (3.9)
Odesa	20 (8.3)	3 (3.4)	17 (11.0)

Abbreviation: OAT, opioid agonist therapy.

^a^ Agreed to the question: “Do you know someone in your personal life (not a patient), who has a substance use disorder (opiate or other)?”

The baseline values of the direct provider sample were higher in all feeling thermometer scales, and lower in stigma and attitude scales compared to the indirect providers. For the direct provider sample, there were no significant changes in findings from the feeling thermometers towards any of the groups before and after intervention. All CAS subscales showed a significant decrease (i.e., improvement) over the baseline‐24 months period: being tough‐minded about addiction (*p* = 0.002), abstinence versus maintenance (*p*<0.001), negative opinions (*p* = 0.006) and incorrect medical information (*p* = 0.004). The abstinence versus maintenance orientation subscale scores also decreased significantly over the baseline‐12 months period (*p*<0.001). The score of the stigma subscale relating to fear of patients who use drugs decreased over the baseline‐24 months period (*p* = 0.007), while the stereotypes subscale score decreased significantly over both the baseline‐12 months (*p* = 0.025) and baseline‐24 months (*p* = 0.002). Finally, resistance to change decreased significantly over baseline‐12 months (*p* = 0.002) (Table 2).

**Table 2:** Provider attitude changes over 12 and 24 months

	Direct providers					Indirect providers				
	Baseline	Baseline–12 months		12–24 months		Baseline	Baseline–12 months		12–24 months	
Measure	Mean (SD)	change in mean	*p*	change in mean	*p*	Mean (SD)	change in mean	*p*	change in mean	*p*
***Feelings thermometer (0‐‐100)***										
Patients who inject drugs	50.1 (19.6)	−1.57	0.633	4.37	0.186	32.2 (19.8)	**5.10**	**0.021**	**9.11**	**<0.001**
Patients with HIV	66.2 (16.1)	2.53	0.339	4.56	0.085	57.3 (14.3)	1.92	0.227	2.88	0.069
Men who have sex with men	43.5 (21.3)	0.87	0.753	2.76	0.321	32.6 (20.7)	3.34	0.081	**4.28**	**0.026**
Women who engage in sex work	45.4 (20.2)	2.54	0.315	2.40	0.342	32.5 (19.9)	2.14	0.240	**6.82**	**<0.001**
Recently released prisoners	51.9 (13.5)	−0.91	0.683	0.25	0.909	45.0 (13.5)	1.75	0.259	**4.74**	**0.002**
***Counsellor Assessment Screen (1‐‐5)***										
Being tough‐minded about addiction	2.75 (0.365)	−0.01	0.811	−**0.14**	**0.002**	3.07 (0.389)	−0.06	0.094	−**0.10**	**0.004**
Abstinence/maintenance orientation	2.87 (0.359)	−**0.22**	**<0.001**	−**0.29**	**<0.001**	3.24 (0.336)	−0.02	0.489	−0.03	0.405
Negative opinions about patients	2.58 (0.332)	−0.09	0.117	−**0.16**	**0.006**	2.80 (0.388)	−0.07	0.100	−**0.12**	**0.002**
Incorrect medical information	2.59 (0.433)	−0.15	0.847	−**0.21**	**0.004**	2.93 (0.523)	−0.10	0.086	−**0.12**	**0.044**
***Stigma Scale (1‐‐5)***										
Intention to discriminate	1.86 (0.430)	−0.02	0.795	−0.02	0.761	2.31 (0.561)	−**0.18**	**0.001**	−**0.19**	**0.001**
Prejudice	2.38 (0.440)	0.07	0.307	0.12	0.068	2.80 (0.509)	−**0.11**	**0.016**	−**0.19**	**<0.001**
Internal shame	2.38 (0.540)	−0.02	0.813	−0.05	0.478	2.82 (0.620)	−**0.20**	**0.002**	−**0.17**	**0.007**
Fear	2.53 (0.575)	−0.08	0.354	−**0.23**	**0.007**	2.92 (0.686)	−**0.20**	**0.002**	−**0.22**	**0.001**
Stereotypes	3.41 (0.487)	−**0.16**	**0.025**	−**0.22**	**0.002**	3.53 (0.549)	−0.08	0.182	−0.02	0.712
***Resistance to change (1‐‐5)***	2.41 (0.354)	−**0.16**	**0.002**	−0.06	0.241	2.61 (0.391)	−**0.21**	**<0.001**	−**0.25**	**<0.001**

All variables that are significant at alpha = 0.05 are bold.

In the indirect provider sample, there was a significant improvement in attitudes observed using feeling thermometers relating to PWID, patients with HIV, MSM, women who engage in sex work and released prisoners over 24‐month observation period. The largest improvement was detected towards PWID with the mean score change of 9.11 points (*p*<0.001). Attitudes towards PWID also improved over the baseline‐12 months period (*p* = 0.021). CAS subscale scores of tough‐mindedness, negative opinions and incorrect medical information improved (i.e., decreased) significantly over the baseline‐24‐month period (*p* = 0.004, *p* = 0.002 and *p* = 0.044, respectively). The only subscale from CAS that did not change significantly was the one assessing abstinence versus maintenance orientation. Intention to discriminate, prejudice, internal shame and fear subscales of the stigma scale showed consistent decreases (i.e., improvements) in scores over both follow‐up periods (Table 2). Stereotype subscales did not display any significant changes. Resistance to change decreased over the total follow‐up period (*p*<0.001 in each follow‐up period).

In a sub‐analysis of change in stigma scales at each clinic over 24 months, two PCCs with <10 patients on OAT (i.e., Kyiv and Odesa), there were no observed changes in any of the stigma constructs. Among the clinics with more OAT patients (range: 14–61), the changes in stigma scales were variable (entry Table 1).

## 4 DISCUSSION

To our knowledge, this is the first study to examine the impact of the contact hypothesis in both direct and indirect providers of OAT through a structurally engineered strategy to integrate these services into PCCs—rather than in specialty clinics providing OAT. Re‐engineering OAT service delivery from a highly stigmatizing specialty clinic to PCCs where all patients in the community are treated potentially represents a *Behavioural Design Intervention* [44], which involves restructuring the presentation of choice (in this case, where patients are treated) in order to change the decision‐making process. In other words, behavioural design interventions make it easier for individuals to make certain choices. For example, prioritizing healthy food choice selection in a location where it is visible and easily accessible and placing less health options elsewhere can be part of a behavioural design intervention to promote healthy eating.

In the present case, only direct OAT providers in PCCs are introduced to specialty treatment using training, choice architecture and framing delivered through Project ECHO. Such a change in delivery is a values‐based approach that seeks to promote ethical behaviours and attitudes within a clinical context [45, 46]. Behavioural design interventions have mostly been used in marketing and more recently to constrain opportunities for sexism [47]. Here, the behavioural design change is placing OAT in a potentially less stigmatizing location (i.e., PCCs) and promoting and supporting PCCs through Project ECHO to deliver guideline‐concordant treatment to a group of PWID who are generally highly stigmatized in healthcare.

Of note, both direct and indirect providers of services to PWID at PCCs prospectively had improvements in their attitudes and lower stigma towards PWID and OAT. For indirect providers, they had contact to PWID through at least two sources: (1) seeing PWID entering the clinic daily to receive treatment; and (2) world‐of‐mouth from other providers who either shared positive experiences or no negative experiences (e.g. disruptions to care for other patients). Either way, indirect providers experienced marked improvements in their stigma constructs over 2 years, with most of the improvement happening over the first 12 months. Overall, direct providers of OAT reported more positive feelings than non‐OAT providers towards every key population group, but their attitudes did not change as much over time, perhaps because of a ceiling effect as the OAT providers were selected for their willingness to provide this service. Importantly, however, their contact with PWID did not worsen their stigma constructs towards PWID. Non‐OAT providers’ attitudes improved towards every group except for PWH. As non‐OAT providers were not exposed to Project ECHO activities, they likely improved their stigma scores because they had increased exposure to these patients, as they regularly came to the clinic and expectations of disruption were likely not met. Thus, the re‐engineering of healthcare delivery has both direct and indirect effects over time, driven primarily by the contact hypothesis, on attitudes towards these patients and the treatments they receive.

Even though there is ample evidence of high stigma towards PWID among providers, there are very few studies exploring stigma reduction among this group. This study specifically examined providers’ attitude change towards PWID and other marginalized groups in a setting where indirect providers did not receive any kind of anti‐stigma training or clinical interventions; direct providers only received tele‐education to enhance clinical skills with no specific stigma‐reduction elements. The results show a significant effect of contact with the marginalized groups decreasing overall stigma and improving attitudes, consistent with our hypothesis. In 1954, *Allport and colleagues* proposed the contact hypothesis: that social interaction between groups is sufficient to reduce negative attitudes [22]. Since then, there have been several studies documenting the effectiveness of contact interventions to reduce stigma in healthcare settings, including both one‐time or prolonged exposure to members of the stigmatized groups. Our study is unique as contact in this case was through a structural intervention that facilitated the integration of OAT into primary care settings, as opposed to providing a designated anti‐stigma intervention (e.g. workshop, placement of students in mental health facilities, etc.). While the effectiveness and meaningfulness of such interventions should not be dismissed, our findings pave the way to the idea that in the settings where time and resources are limited, and anti‐stigma interventions cannot be worked into the planning of the project, integration of care of marginalized populations (PWID, PWH, TB, etc.) in primary health centres, along with the positive effects on health outcomes [48, 49, 50, 51, 52], can be an efficient strategy to reduce provider stigma, even among indirect providers, and improve future intergroup interactions [29].

Although organizational and institutional factors are important drivers of stigma [29, 53], there are very few studies that examine service integration and policy change effect on attitudes [54, 55]. Here, the integration of OAT services in PCCs was a complex and multilevel process. Because OAT adoption in PCCs in Ukraine was unprecedented, PCCs underwent major organizational and policy change. In addition to policy changes, PCCs adapted evidence based practice and introduced a new model of service provision. These organizational changes, and general organizational openness towards OAT, could be a contributor to the overall improved attitudes among all employees. These factors may also explain the reduction in resistance to organizational change in both groups of providers as a new EBP was introduced (i.e., OAT). Attitudes and perceptions were higher overall among direct OAT providers at baseline meaning that this group had positive attitudes towards PWID and other groups from the outset. As a result, interpersonal relationships and peer‐influence could have also contributed to the outcomes as direct OAT providers may have shared their own positive experiences with PWID.

Those who were direct providers expressed willingness and readiness to work with PWID at baseline, a self‐selection factor that can potentially explain overall better attitudes in this group and a relatively lower degree of change. OAT providers, however, experienced significant improvements in their attitudes towards OAT. This may, in part, be explained by their observation that the patients they cared for, experienced clinical improvements over time. Contrary to available evidence, abstinence has previously been regarded in Eastern Europe and Central Asia as an SOC for PWID, yet OAT providers here potentially observed that OAT is highly effective relative to abstinence therapies based on their observations, thereby influencing their improvements in attitudes.

Here, contact between patients and providers, or among different types of providers, was not quantified, and we cannot determine if the intensity or length has a significant effect of attitude change. Levels of contact are unknown, and future research is needed to determine what types of intergroup contact and what duration would be most beneficial for stigma reduction among provider groups.

## 5 CONCLUSIONS

Integrating OAT services within primary settings in Ukraine potentially represents a behavioural design intervention and was associated with significantly reduced stigma and improved attitudes towards PWID, possibly through increased intergroup contact between PWID and general clinical staff. Multilevel interventions such as integration of specialized services in PCCs decrease stigma at multiple different levels, and, in addition to anticipated positive effects on health outcomes, can improve provider attitudes. The results from Ukraine suggest that behavioural design interventions may be a potent stigma reduction strategy and re‐engineering OAT services by integrating them into PCCs is one way to reduce stigma towards PWID in healthcare settings, including in low and middle income countries and other Eastern European and Central Asian countries where stigma towards PWID is high and disrupts care.

## COMPETING INTERESTS

The authors have no competing interests.

## AUTHORS’ CONTRIBUTIONS

DJB and EM conducted all analyses and wrote the initial draft of the manuscript with input from all other authors. LMM, KD, KL, VAE, IP, MF and MSH were responsible for data collection. FLA and SD supervised all data collection and analysis. All authors reviewed and approved the final manuscript.

## Supporting information

Supporting information

## Data Availability

As these data are part of a larger and ongoing study, the data that support the findings of this study are available from the corresponding author upon reasonable request.

## References

[1] Heijnders M , Van Der Meij S . The fight against stigma: an overview of stigma‐reduction strategies and interventions. Psychol Health Med. 2006;11(3):353–363. 17130071 10.1080/13548500600595327

[2] Rinehart R , Rao D , Amico RK , Ruiz E , Brandes P , Correa C , et al. Experienced HIV‐related stigma and psychological distress in Peruvian sexual and gender minorities: a longitudinal study to explore mediating roles of internalized HIV‐related stigma and coping styles. AIDS Behav. 2019;23:661–674. 30506474 10.1007/s10461-018-2348-2 PMC6408311

[3] Chambers SK , Dunn J , Occhipinti S , Hughes S , Baade P , Sinclair S , et al. A systematic review of the impact of stigma and nihilism on lung cancer outcomes. BMC Cancer. 2012;12(1):184. 22607085 10.1186/1471-2407-12-184 PMC3517321

[4] Kane JC , Elafros MA , Murray SM , Mitchell EMH , Augustinavicius JL , Causevic S , et al. A scoping review of health‐related stigma outcomes for high‐burden diseases in low‐ and middle‐income countries. BMC Med. 2019;17(1):17. 30764819 10.1186/s12916-019-1250-8 PMC6376728

[5] Remien RH , Bauman LJ , Mantell JE , Tsoi B , Lopez‐Rios J , Chhabra R , et al. Barriers and facilitators to engagement of vulnerable populations in HIV primary care in New York City. J Acquir Immune Defic Syndr. 2015;69 Suppl 1(0 1):S16–S24. 25867774 10.1097/QAI.0000000000000577 PMC4559146

[6] Corrigan P . How stigma interferes with mental health care. Am Psychol. 2004;59(7):614–625. 15491256 10.1037/0003-066X.59.7.614

[7] Henderson C , Noblett J , Parke H , Clement S , Caffrey A , Gale‐Grant O , et al. Mental health‐related stigma in health care and mental health‐care settings. Lancet Psychiatry. 2014;1(6):467–482. 26361202 10.1016/S2215-0366(14)00023-6

[8] Luo S , Lin C , Feng N , Wu Z , Li L . Stigma towards people who use drugs: a case vignette study in methadone maintenance treatment clinics in China. Int J Drug Policy. 2019;71:73–77. 31233972 10.1016/j.drugpo.2019.06.005 PMC6868318

[9] Biancarelli DL , Biello KB , Childs E , Drainoni M , Salhaney P , Edeza A , et al. Strategies used by people who inject drugs to avoid stigma in healthcare settings. Drug Alcohol Depend. 2019;198:80–86. 30884432 10.1016/j.drugalcdep.2019.01.037 PMC6521691

[10] Muncan B , Walters SM , Ezell J , Ompad DC . “They look at us like junkies”: influences of drug use stigma on the healthcare engagement of people who inject drugs in New York City. Harm Reduct J. 2020;17(1):53. 32736624 10.1186/s12954-020-00399-8 PMC7393740

[11] Degenhardt L , Grebely J , Stone J , Hickman M , Vickerman P , Marshall BDL , et al. Global patterns of opioid use and dependence: harms to populations, interventions, and future action. Lancet. 2019;394(10208):1560–1579. 31657732 10.1016/S0140-6736(19)32229-9 PMC7068135

[12] Mazhnaya A , Marcus R , Bojko MJ , Zelenev A , Makarenko I , Pykalo I , et al. Opioid agonist treatment and improved outcomes at each stage of the HIV treatment cascade in people who inject drugs in Ukraine. J Acquir Immune Defic Syndr. 2018;79(3):288–295. 30312275 10.1097/QAI.0000000000001827 PMC8215524

[13] Low AJ , Mburu G , Welton NJ , May MT , Davies CF , French C , et al. Impact of opioid substitution therapy on antiretroviral therapy outcomes: a systematic review and meta‐analysis. Clin Infect Dis. 2016;63(8):1094–1104. 27343545 10.1093/cid/ciw416 PMC5036913

[14] Haddad MS , Zelenev A , Altice FL . Buprenorphine maintenance treatment retention improves nationally recommended preventive primary care screenings when integrated into urban federally qualified health centers. J Urban Health. 2015;92(1):193–213. 25550126 10.1007/s11524-014-9924-1 PMC4338126

[15] Haddad MS , Zelenev A , Altice FL . Integrating buprenorphine maintenance therapy into federally qualified health centers: real‐world substance abuse treatment outcomes. Drug Alcohol Depend. 2013;131(1–2):127–135. 23332439 10.1016/j.drugalcdep.2012.12.008 PMC3674170

[16] Cioe K , Biondi BE , Easly R , Simard A , Zheng X , Springer SA . A systematic review of patients' and providers' perspectives of medications for treatment of opioid use disorder. J Subst Abuse Treat. 2020;119:108146. 33138929 10.1016/j.jsat.2020.108146 PMC7609980

[17] Madden EF , Prevedel S , Light T , Sulzer SH . Intervention stigma toward medications for opioid use disorder: a systematic review. Subst Use Misuse. 2021;56(14):2181–2201. 34538213 10.1080/10826084.2021.1975749

[18] Vijay A , Earnshaw VA , Tee YC , Pillai V , White Hughto JM , Clark K , et al. Factors associated with medical doctors' intentions to discriminate against transgender patients in Kuala Lumpur, Malaysia. LGBT Health. 2018;5(1):61–68. 29227183 10.1089/lgbt.2017.0092 PMC5770086

[19] Stangl AL , Earnshaw VA , Logie CH , Van Brakel W , C Simbayi L , Barré I , et al. The Health Stigma and Discrimination Framework: a global, crosscutting framework to inform research, intervention development, and policy on health‐related stigmas. BMC Med. 2019;17(1):31. 30764826 10.1186/s12916-019-1271-3 PMC6376797

[20] Livingston JD , Milne T , Fang ML , Amari E . The effectiveness of interventions for reducing stigma related to substance use disorders: a systematic review. Addiction. 2012;107(1):39–50. 10.1111/j.1360-0443.2011.03601.x PMC3272222 21815959

[21] Kolodziej ME , Johnson BT . Interpersonal contact and acceptance of persons with psychiatric disorders: a research synthesis. J Consult Clin Psychol. 1996;64(6):1387–1396. 8991325 10.1037//0022-006x.64.6.1387

[22] Allport GW , Clark K , Pettigrew T . The nature of prejudice. 1954.

[23] Pettigrew TF , Tropp LR . A meta‐analytic test of intergroup contact theory. J Pers Soc Psychol. 2006;90(5):751–783. 16737372 10.1037/0022-3514.90.5.751

[24] Pettigrew TF , Tropp LR , Wagner U , Christ O . Recent advances in intergroup contact theory. Int J Intercult Relat. 2011;35:271–280.

[25] Dovidio JF , Gaertner SL , Kawakami K . Intergroup contact: the past, present, and the future. Group Process Intergroup Relat. 2003;6:5–20.

[26] Economou M , Kontoangelos K , Peppou LE , Arvaniti A , Samakouri M , Douzenis A , et al. Medical students' attitudes to mental illnesses and to psychiatry before and after the psychiatric clerkship: training in a specialty and a general hospital. Psychiatry Res. 2017;258:108–115. 28992547 10.1016/j.psychres.2017.10.009

[27] Hatzenbuehler ML , Phelan JC , Link BG . Stigma as a fundamental cause of population health inequalities. Am J Public Health. 2013;103(5):813–821. 23488505 10.2105/AJPH.2012.301069 PMC3682466

[28] Rao D , Elshafei A , Nguyen M , Hatzenbuehler ML , Frey S , Go VF . A systematic review of multi‐level stigma interventions: state of the science and future directions. BMC Med. 2019;17(1):41. 30770756 10.1186/s12916-018-1244-y PMC6377735

[29] Cook JE , Purdie‐Vaughns V , Meyer IH , Busch JTA . Intervening within and across levels: a multilevel approach to stigma and public health. Soc Sci Med. 2014;103:101–109. 24513229 10.1016/j.socscimed.2013.09.023

[30] Morozova O , Dvoriak S , Pykalo I , Altice FL . Primary healthcare‐based integrated care with opioid agonist treatment: first experience from Ukraine. Drug Alcohol Depend. 2017;173:132–138. 28242537 10.1016/j.drugalcdep.2016.12.025 PMC5545131

[31] Scott JD , Unruh KT , Catlin MC , Merrill JO , Tauben DJ , Rosenblatt R , et al. Project ECHO: a model for complex, chronic care in the Pacific Northwest region of the United States. J Telemed Telecare. 2012;18(8):481–484. 23209269 10.1258/jtt.2012.GTH113 PMC3647034

[32] Arora S , Kalishman S , Thornton K , Dion D , Murata G , Deming P , et al. Expanding access to hepatitis C virus treatment–Extension for Community Healthcare Outcomes (ECHO) project: disruptive innovation in specialty care. Hepatology. 2010;52(3):1124–1133. 20607688 10.1002/hep.23802 PMC3795614

[33] Arora S , Kalishman S , Dion D , Som D , Thornton K , Bankhurst A , et al. Partnering urban academic medical centers and rural primary care clinicians to provide complex chronic disease care. Health Aff (Millwood). 2011;30(6):1176–1184. 21596757 10.1377/hlthaff.2011.0278 PMC3856208

[34] Walters SM , Li WP , Saifi R , Azwa I , Syed Omar SF , Collier ZK , et al. Barriers and facilitators to implementing Project ECHO in Malaysia during the COVID‐19 pandemic. J Int Assoc Provid AIDS Care. 2022;21. 10.1177/23259582221128512 PMC9528038 36177542

[35] Earnshaw VA , Jin H , Wickersham J , Kamarulzaman A , John J , Altice FL . Exploring intentions to discriminate against patients living with HIV/AIDS among future healthcare providers in Malaysia. Trop Med Int Health. 2014;19(6):672–679. 24666546 10.1111/tmi.12306 PMC4450358

[36] Earnshaw VA , Jin H , Wickersham JA , Kamarulzaman A , John J , Lim SH , et al. Stigma toward men who have sex with men among future healthcare providers in Malaysia: would more interpersonal contact reduce prejudice? AIDS Behav. 2016;20(1):98–106. 26324078 10.1007/s10461-015-1168-x PMC4718796

[37] Jin H , Earnshaw VA , Wickersham JA , Kamarulzaman A , Desai MM , John J , et al. An assessment of health‐care students' attitudes toward patients with or at high risk for HIV: implications for education and cultural competency. AIDS Care. 2014;26(10):1223–1228. 24625279 10.1080/09540121.2014.894616 PMC4089975

[38] Tee YC , Earnshaw VA , Altice FL , Jin H , Kamarulzaman A , Wickersham JA . Evaluating physicians' intention to discriminate against patients living with HIV in Malaysia. AIDS Behav. 2019;23(4):1039–1047. 30560483 10.1007/s10461-018-2362-4 PMC6459712

[39] Alwin DF . Feeling thermometers versus 7‐point scales. Sociol Methods Res. 1997;25(3):318–340.

[40] Kang S‐Y , Magura S , Nwakeze P , Demsky S . Counselor attitudes in methadone maintenance. J Main Addict. 1998;1(2):41–58.

[41] Stein JA , Li L . Measuring HIV‐related stigma among Chinese service providers: confirmatory factor analysis of a multidimensional scale. AIDS Behav. 2008;12(5):789–795. 18064554 10.1007/s10461-007-9339-z PMC2700338

[42] Ni Z , Shrestha R , Earnshaw VA , Tee YC , Altice FL , Azwa I , et al. Exploring Malaysian physicians' intention to discriminate against gay, bisexual, and other men who have sex with men patients. LGBT Health. 2022;10(2):169–175. 36251945 10.1089/lgbt.2021.0452 PMC9986015

[43] Oreg S . Resistance to change: developing an individual differences measure. J Appl Psychol. 2003;88:680–693. 12940408 10.1037/0021-9010.88.4.680

[44] Norman DA . The psychology of everyday things. Basic Books; 1988.

[45] Niedderer K , Ludden G , Clune SJ , Lockton D , Mackrill J , Morris A , et al. Design for behaviour change as a driver for sustainable innovation: challenges and opportunities for implementation in the private and public sectors. Int J Des. 2016;10(2):67–85.

[46] Niedderer K , MacKrill J , Clune SJ , Evans M , Lockton D , Ludden G , et al. Joining forces: investigating the influence of design for behavior change on sustainable innovation. 2014:620–630.

[47] Bohnet I . What works: gender equality by design. Cambridge, MA: Harvard University Press; 2016.

[48] Pashchenko O , Bromberg DJ , Dumchev K , Lamonaca K , Pykalo I , Filippovych M , et al. Preliminary analysis of self‐reported quality health indicators of patients on opioid agonist therapy at specialty and primary care clinics in Ukraine: a randomized control trial. PLoS Glob Public Health. 2022;2(11):e0000344. 36962514 10.1371/journal.pgph.0000344 PMC10021202

[49] Haskins L , Chiliza J , Barker P , Connolly C , Phakathi S , Feeley A , et al. Evaluation of the effectiveness of a quality improvement intervention to support integration of maternal, child and HIV care in primary health care facilities in South Africa. BMC Public Health. 2020;20(1):318. 32164597 10.1186/s12889-020-8397-2 PMC7069172

[50] Solomon SS , Solomon S , McFall AM , Srikrishnan AK , Anand S , Verma V , et al. Integrated HIV testing, prevention, and treatment intervention for key populations in India: a cluster‐randomised trial. Lancet HIV. 2019;6(5):e283–e296. 30952565 10.1016/S2352-3018(19)30034-7 PMC6524776

[51] Tun NN , McLean A , Wilkins E , Hlaing M , Aung Y , Linn T , et al. Integration of HIV services with primary care in Yangon, Myanmar: a retrospective cohort analysis. HIV Med. 2020;21(9):547–556. 32687684 10.1111/hiv.12886

[52] Butler M , Kane RL , McAlpine D , Kathol RG , Fu SS , Hagedorn H , et al. Integration of mental health/substance abuse and primary care. Evid Rep Technol Assess (Full Rep). 2008:1–362. PMC4781124 19408966

[53] Hatzenbuehler ML . Structural stigma: research evidence and implications for psychological science. Am Psychol. 2016;71(8):742–751. 27977256 10.1037/amp0000068 PMC5172391

[54] Li L , Wu Z , Liang LJ , Lin C , Zhang L , Guo S , et al. An intervention targeting service providers and clients for methadone maintenance treatment in China: a cluster‐randomized trial. Addiction. 2013;108(2):356–366. 22788780 10.1111/j.1360-0443.2012.04020.x PMC3483381

[55] Pulerwitz J , Oanh KT , Akinwolemiwa D , Ashburn K , Nyblade L . Improving hospital‐based quality of care by reducing HIV‐related stigma: evaluation results from Vietnam. AIDS Behav. 2015;19(2):246–256. 25382350 10.1007/s10461-014-0935-4

