# Supplementary material for: Integrating methadone into primary care settings in Ukraine: effects on provider stigma and knowledge
Source: J Int AIDS Soc. 2024 Feb 20;27(2):e26202. doi: 10.1002/jia2.26202 (PMC10879646; doi:10.1002/jia2.26202)
Supplement: Supplementary file 1 — Supporting information [file JIA2-27-e26202-s001.docx]

*Supplementary Table 1. Changes in mean scores in stigma scales of discrimination, prejudice, shame, fear, and stereotypes in each clinic over 24 months.*

| **Clinic** | **N of OAT patients** | **Discrimination** | | **Prejudice** | | **Shame** | | **Fear** | | **Stereotypes** | |
| --- | --- | --- | --- | --- | --- | --- | --- | --- | --- | --- | --- |
|  |  | **Mean Change** | **P** | **Mean Change** | **P** | **Mean Change** | **P** | **Mean Change** | **P** | **Mean Change** | **P** |
| **Rivne** |  |  |  |  |  |  |  |  |  |  |  |
| PCC | 38 | -0.32 | 0.088 | -0.08 | 0.582 | **-0.4** | **0.027** | **-0.54** | **0.001** | -0.22 | 0.214 |
| PCC+P4P | 30 | -0.36 | 0.073 | **-0.43** | **0.012** | -0.32 | 0.065 | -0.29 | 0.066 | **-0.26** | **0.016** |
| **Kramatorsk** |  |  |  |  |  |  |  |  |  |  |  |
| PCC | 61 | -0.09 | 0.666 | -0.1 | 0.468 | -0.12 | 0.417 | 0.16 | 0.456 | -0.04 | 0.796 |
| PCC+P4P | 42 | -0.01 | 0.958 | -0.09 | 0.149 | -0.05 | 0.629 | -0.01 | 0.950 | 0.04 | 0.657 |
| **Cherkassy** |  |  |  |  |  |  |  |  |  |  |  |
| PCC | 43 | 0.01 | 0.929 | -0.16 | 0.232 | -0.19 | 0.376 | **-0.63** | **0.010** | **-0.46** | **0.009** |
| PCC+P4P | 33 | 0.01 | 0.962 | -0.32 | 0.179 | **-0.62** | **0.011** | **-0.86** | **0.010** | -0.19 | 0.232 |
| **Odesa** |  |  |  |  |  |  |  |  |  |  |  |
| PCC | 22 | -0.45 | 0.135 | **-0.62** | **0.018** | **-0.45** | **0.004** | -0.44 | 0.217 | **-0.56** | **0.004** |
| PCC+P4P | 8 | -0.2 | 0.345 | -0.01 | 0.975 | 0.09 | 0.677 | -0.15 | 0.600 | 0.19 | 0.232 |
| **Dnipro** |  |  |  |  |  |  |  |  |  |  |  |
| PCC | 43 | **-0.43** | **0.047** | -0.32 | 0.148 | **-0.87** | **<0.001** | **-0.85** | **0.006** | **-0.34** | **0.017** |
| PCC+P4P | 40 | 0.28 | 0.395 | -0.08 | 0.626 | -0.5 | 0.097 | **-0.81** | **0.005** | -0.15 | 0.474 |
| **Zhytomyr** |  |  |  |  |  |  |  |  |  |  |  |
| PCC | 47 | **-0.38** | **<0.001** | -0.46 | 0.200 | **-1.01** | **0.011** | -0.98 | 0.069 | 0.25 | 0.574 |
| PCC+P4P | 34 | **-0.81** | **0.006** | -0.05 | 0.776 | -0.11 | 0.674 | -0.43 | 0.178 | 0.14 | 0.611 |
| **Slovyansk** |  |  |  |  |  |  |  |  |  |  |  |
| PCC | 33 | -0.03 | 0.886 | 0.12 | 0.525 | 0.03 | 0.808 | 0.24 | 0.131 | 0.02 | 0.853 |
| PCC+P4P | 26 | 0.09 | 0.371 | -0.14 | 0.494 | -0.01 | 0.927 | -0.18 | 0.186 | -0.08 | 0.629 |
| **Kyiv** |  |  |  |  |  |  |  |  |  |  |  |
| PCC | 14 | 0.44 | 0.084 | -0.05 | 0.777 | 0.2 | 0.331 | **-0.4** | **0.027** | -0.31 | 0.085 |
| PCC+P4P | 8 | -0.11 | 0.083 | -0.49 | 0.08 | -0.37 | 0.078 | -0.07 | 0.721 | 0.09 | 0.616 |
| **Kropyvnytskyi** |  |  |  |  |  |  |  |  |  |  |  |
| PCC | 34 | -0.12 | 0.671 | 0.37 | 0.215 | 0.35 | 0.266 | 0.18 | 0.630 | -0.09 | 0.508 |
| PCC+P4P | 42 | **0.35** | **0.004** | 0.13 | 0.319 | 0.33 | 0.153 | 0.13 | 0.554 | 0.1 | 0.571 |
| **Kryvyi** **Rig** |  |  |  |  |  |  |  |  |  |  |  |
| PCC | 46 | -0.09 | 0.508 | 0.18 | 0.148 | -0.13 | 0.563 | -0.29 | 0.126 | 0.24 | 0.178 |
| PCC+P4P | 49 | 0.08 | 0.696 | -0.03 | 0.880 | -0.02 | 0.911 | -0.3 | 0.088 | -0.23 | 0.130 |
| **Mariupol** |  |  |  |  |  |  |  |  |  |  |  |
| PCC | 29 | -0.11 | 0.473 | 0.12 | 0.444 | -0.27 | 0.168 | -0.16 | 0.19 | **-0.31** | **0.043** |
| PCC+P4P | 43 | -0.17 | 0.174 | -0.08 | 0.623 | 0.04 | 0.837 | -0.3 | 0.075 | -0.09 | 0.476 |
| **Mykolaiv** |  |  |  |  |  |  |  |  |  |  |  |
| PCC | 43 | -0.06 | 0.637 | **-0.22** | **0.025** | -0.2 | 0.192 | 0.12 | 0.451 | -0.07 | 0.576 |
| PCC+P4P | 46 | -0.02 | 0.873 | **0.02** | **0.828** | **0.29** | **0.009** | -0.1 | 0.581 | 0.04 | 0.782 |

Abbr.: OAT Opioid Agonist Therapies; PCC Primary care clinic; P4P pay-for-performance.
